# Baseline High Viral Load and Unfavorable Patterns of Alanine Aminotransferase Change Predict Virological Relapse in Patients With Chronic Hepatitis C Genotype 1 or 2 Obtaining Rapid Virological Response During Antiviral Therapy

**DOI:** 10.5812/hepatmon.11892

**Published:** 2013-10-21

**Authors:** Kung-Hung Lin, Hsien-Chung Yu, Ping-I Hsu, Wei-Lun Tsai, Wen-Chi Chen, Chun-Ku Lin, Hoi-Hung Chan, Fong-Wei Tsay, Kwok-Hung Lai

**Affiliations:** 1 Division of Gastroenterology, Department of Internal Medicine, Kaohsiung Veterans General Hospital, Kaohsiung, Taiwan; 2 Physical Examination Center, Kaohsiung Veterans General Hospital, Kaohsiung, Taiwan

**Keywords:** Chronic Hepatitis C, Pegylated Interferon Alfa-2a, Ribavirin, Relapse

## Abstract

**Background:**

Rapid virological response (RVR) strongly predicts sustained virological response (SVR) in patients with chronic hepatitis C (CHC), and abbreviates antiviral therapy in some patients.

**Objectives:**

To identify factors predicting virological relapse (VR) in CHC patients who attained RVR.

**Patients and Methods:**

Medical records of 133 CHC patients with an RVR after completing 24 weeks of antiviral therapy (a combination of pegylated interferon-α and ribavirin) were analyzed. Baseline characteristics and on-treatment responses were compared between the patients with an SVR and those with VR. Patients with normal alanine aminotransferase (ALT) levels at weeks 4 and 12 and at the end-of-treatment (EoT) and patients with elevated, but constantly decreasing, ALT levels were classified as having favorable patterns of ALT change. A trend of increasing ALT levels either between weeks 4 and 12 or between weeks 12 and EoT was classified as unfavorable. A high viral load (HVL) was defined as a baseline HCV RNA ≥ 600000 IU/mL.

**Results:**

In total, 116 (87.2%) patients had a SVR and 14 (10.5%) had VR. The VR rates were comparable between patients with genotype-1 (13.1%) and genotype-2 infection (8.7%) (P = 0.572). Multivariate analysis revealed that HVL (P = 0.015; odds ratio [OR] = 14.754; 95% confidence interval (CI) = 1.671–130.240), and unfavorable ALT patterns (P = 0.039; OR = 4.397; 95% CI = 1.078–17.930) independently predicted VR. In subgroup analysis, low viral load (LVL) patients had a minimal VR rate (1.8%). Among the HVL patients, the VR rate of those using peg-IFN-α-2a was relatively low (9.1%). Patients using peg-IFN-α-2b had a slightly higher VR rate (23.8%; P = 0.128), and patients with favorable patterns of ALT changes had a lower VR rate (10.3%) compared to the 53.8% in patients with unfavorable ALT patterns (P = 0.005).

**Conclusions:**

In southern Taiwan, 24 weeks of antiviral therapy achieved a high SVR rate in patients with CHC attaining RVR, except in the subgroup of patients treated with peg-IFN-α-2b with HVL and on-treatment unfavorable ALT patterns.

## 1. Background

The currently recommended therapy for chronic hepatitis C virus (HCV) infection is a combination of pegylated interferon-α (peg-IFN-α) and ribavirin (RBV) (1, 2). Achieving a rapid virologic response (RVR, defined as a undetectable serum HCV RNA level at week 4 of treatment) is highly predictive of attaining a sustained virologic response (SVR, defined a undetectable serum HCV RNA 24 weeks after cessation of treatment), regardless of genotype and the treatment regimen (3, 4). Several studies have also reported high SVR rates (76%–89% in genotype-1, and 80%–98% in genotype-2/3) in patients attaining an RVR, even when abbreviated therapies were administered (3, 5-12). Therefore, current practice guidelines suggest that the standard 48-week (for genotype-1/4 HCV infection) or 24-week course (for genotype-2/3) can be abbreviated to 24 or 16 weeks, respectively, for patients obtaining an RVR, if the patient has a low baseline viral load (< 400000–800000 IU/mL) (1, 2). Relapse is uncommon among patients attaining RVR, and factors other than baseline viral load that predict relapse have been reported rarely. Such predictive factors need to be identified to recognize patients in which 48-week courses are more likely to avoid relapse and attain an SVR. A variety of baseline and on-treatment factors have been reported to predispose relapse in patients undergoing standard 48-week (genotype-1) or 24-week (genotype 2/3) combination therapies (13-21).

## 2. Objectives

The aim of this study was to identify factors that could predict virologic relapse (VR) in patients with CHC attaining RVR during the combination therapy.

## 3. Patients and Methods

Medical records of patients with HCV infection who received peg-IFN-α and RBV at the Kaohsiung Veterans General Hospital in Taiwan between Nov 2009 and Oct 2011 were retrospectively reviewed. All patients seropositive for anti-HCV antibody (determined using Ax SYM HCV 3.0; Abbott Laboratories, Wiesbaden-Delkenheim, Germany), had detectable HCV-RNA values (measured by Cobas TaqMan HCV assay, Roche Diagnostics, Indianapolis, IN, The USA, with a lower detection limit of 25 IU/mL), and ALT levels >40 IU/L. Exclusion criteria included concomitant human immunodeficiency virus infection, and hepatitis due to reasons other than hepatitis C (e.g. autoimmune hepatitis, hemochromatosis, and Wilson’s disease). Patients with concomitant hepatitis B virus infection were not excluded because those with dual infection respond as well as patients with only HCV (22). Patients with heavy alcohol intake, defined as habitual drinking with daily alcohol consumption > 50 g, were included only if they suspended alcohol intake completely or restricted its use to occasional drink before and throughout the combination therapy (2). This study was approved by the Institutional Review Board (IRB) of the Kaohsiung Veterans General Hospital (IRB: VGHKS12-CT6-01).

### 3.1. Treatment and Follow-Up

All patients received free combination therapy consisting of RBV and either peg-IFN-α-2a (Pegasys, Roche, Basel, Switzerland) or peg-IFN-α-2b (PEG-Intron; Schering-Plough, Kenilworth, NJ, The USA) in accordance with the regulations of the National Health Insurance Administration, Taiwan (23). For patients attaining a RVR, the duration of treatment was restricted to 24 weeks, regardless of genotype. For patients without a RVR, the 48-week course was offered. For those who failed to achieve undetectable HCV-RNA or failed to attain ≥2log decrease of HCV-RNA at week 12 (i.e. failed to attain an EVR), therapy was discontinued. The choice of peg-IFN-α-2a or peg-IFN-α-2b was not randomized, but was made at the discretion of the treating physician. Peg-IFN-α and RBV were administered at doses in accordance with the current standard of care (1, 2).

Baseline complete blood cell counts and differential counts, liver function tests, HCV-RNA levels, genotypes (by a 5’ noncoding region- and core-based reverse transcriptase PCR assay with sequencing (24), and sonographic results were obtained in all patients). Pretreatment liver biopsy was not mandatory. It was offered to individuals as a part of the evaluation for diagnosis and prognosis of the disease, in the setting of routine clinical practice. Fibrosis evaluation (scoring by the METAVIR system (25) was available in the patients undergoing liver biopsy). Cirrhosis was diagnosed by ultrasonography, endoscopy (esophageal or gastric varices), or liver biopsy. Fatty liver was diagnosed by ultrasound if liver echogenicity exceeded that of renal cortex. High viral load (HVL) and low viral load (LVL) were defined as baseline HCV-RNA ≥ and <600000 IU/mL, respectively (2). The patterns of ALT change were classified as either favorable or unfavorable based on the study by Basso et al. and determined by comparing ALT levels at weeks 4 (ALT_W4_), 12 (ALT_W12_), and 24 (ALT_W24_) (13). A favorable pattern was defined as the presence of normal ALT_W4_, ALT_W12_, and ALT_W24_ levels. Patients with elevations at one or more time points were generally classified as having unfavorable patterns, except those with constantly decreasing ALT levels (i.e. ALT_W24_ ≤ ALT_W12_ ≤ ALT_W4_), which reflects progressive resolution of hepatic damage. Such patients were therefore classified as having a favorable pattern.

Information regarding adherence to treatment, clinical/hematologic adverse effects, and biochemical response, during the treatment period and after completion of treatment was collected. HCV-RNA levels were obtained to determine RVR, EVR, end-of-treatment (EoT) virologic response (ETVR, defined as undetectable HCV-RNA levels at EoT), virologic breakthrough (defined as reappearance of HCV-RNA in plasma during the combination therapy), virologic relapse (defined as reappearance of plasma HCV-RNA after EoT), and SVR. During the treatment, if significant side effects were occurred, dose reductions or suspension of the treatment were achieved according to the current practical guideline (2). Patients who had received ≥80% of the expected Peg-IFN and RBV doses for ≥80% of its expected duration were regarded as achieving 80/80/80 adherence.

### 3.2. Statistical Analysis

Quantitative parameters were expressed as either median or 25th–75th percentile values or mean ± standard deviation. Categorical variables were compared with χ^2^ and Fisher exact tests. The continuous variables were compared using a Mann-Whitney U test. For identification of factors related to virologic relapse after the combination therapy, multivariate logistic regression analysis was applied. All statistical analyses were based on two-side hypothesis tests with a significance level of P < 0.05. All data analyses were performed using SPSS for Windows (version 12; SPSS Inc., Chicago, IL, USA).

## 4. Results

Medical records of 212 patients were reviewed. Therapy was prematurely discontinued in 25 patients owing to intolerable adverse effects. One patient died due to hypertensive cerebellar hemorrhage. In the 11 patients without either an RVR or an EVR, therapies were terminated according to the regulations of the National Health Insurance Administration, Taiwan. Among the remaining 175 patients, an RVR was attained in 133 (62.7% [95% confidence interval (C.I), 56.2%-69.2%] by intention-to-treat [ITT] analysis, 76% [95% C.I., 69.7%-82.3%] by per-protocol [PP] analysis), and an SVR was achieved in 140 patients (66.0% [95% C.I., 59.6%-72.4%] by ITT, 75.3% [95% C.I., 69.1%-81.5%] by PP analysis). Among the 133 patients attaining an RVR, virologic breakthrough and relapse occurred in 3 and 14 patients, respectively. An SVR was obtained in the remaining 116 patients (87.2% [95% C.I., 81.5%-92.9%] by PP analysis).

Baseline characteristics of the 133 patients achieved an RVR are summarized in Table 1, and the treatment profiles, and virologic responses are present in Table 2 (stratified by genotypes). The baseline characteristics of patients with genotype-1 (G-1) infection were similar to those infected with genotype-2 (G-2). The mean doses of peg-IFN-α (either 2a or 2b) were similar between the G-1 and G-2 patients (P = 0.340 and 0.517, respectively), but the mean ribavirin dose was significantly higher in the patients with G-1 than those with G-2 (15.48 versus 13.90 mg/kg/day, P < 0.001). The 80/80/80 adherence rates were comparable in both groups (92.1% for G-1 and 87.1% for G-2, P = 0.408). The rates of EVR [98.4% (95% C.I., 95.3%-100%) for G-1 versus 100% (95% C.I. 100%-100%) for G-2, P = 0.474], ETVR [96.8% (95% C.I. 92.5%-100%) for G-1 versus 98.6% (95% C.I. 95.8%-100%) for G-2, P = 0.603), and SVR [84.1% (95% C.I. 75.1%-93.1%) for G-1 versus 90% (95% C.I. 83.0%-97.0%) for G-2, P = 0.207) were also comparable between the two groups. Only 8 patients (13.1%, 95% C.I. 4.6%-21.6%) and 6 patients (8.7%, 95% C.I. 2.0%-15.4%) in the G-1 and G-2 groups, respectively, underwent virologic relapse during the 24-week follow-up period.

**Table 1.** Baseline Characteristics of 133 Patients With Chronic Hepatitis C Achieved a Rapid Virologic Response During Antiviral Therapy. Patients were Stratified by Genotype, and Data Are Expressed as Either Median (25th–75th Percentile Values) or Number (Percentage)

Variable	Genotype-1, (n = 63), No. (%)	Genotype-2, (n = 70), No. (%)	P value
**Age, y **	56 (44-64)	56 (46-64)	0.804
**Gender, male/female**	34/29 (54.0/46.0)	36/34 (51.4/48.6)	0.770
**Body mass index, kg/m2**	24.3 (22.6-25.9)	25.3 (22.5-27.5)	0.116
**History of DM ** ^**a**^ **, yes/no**	10/53 (15.9/84.1)	14/56 (20/80)	0.537
**Heavy alcohol intake **^b^** , yes/no**	7/56 (11.1/88.9)	8/62 (11.4/88.6)	0.954
**Albumin, g/dL**	4.3 (4.2-4.5)	4.3 (4.1-4.6)	0.821
**Total bilirubin, mg/dL**	0.6 (0.5-0.9)	0.7 (0.6-0.9)	0.218
**AST ** ^**a**^ **, U/L**	73 (48-118)	73 (50-118)	0.944
**ALT ** ^**a**^ **, U/L**	135 (82-228)	123 (75-263)	0.680
**ALK-p** ^** a**^ **, U/L**	76 (62-101)	79 (67-95)	0.789
**Prothrombin time ^**a**^, INR**	1.01 (0.98-1.05)	1.03 (0.99-1.07)	0.146
**WBC ** ^**a**^ ** counts, /mm3** ****	5710 (5030-6580)	5550 (4888-6763)	0.776
**Hemoglobin, g/dL **	14.4 (13.5-15.4)	14.0 (12.8-15.7)	0.456
**Platelet counts, × 10 ^**3**^ /μL **	184 (145-226)	175 (135-221)	0.593
**AST-platelet ratio index **	0.93 (0.61-1.70)	1.06 (0.64-2.01)	0.654
**Fibrosis stage ^** c**^ , F1/2/3/4 **	1/7/6/2	0/12/2/0	0.150
**Cirrhosis status, yes/no**	6/57 (9.5/90.5)	2/68 (2.9/97.1)	0.149
**Fatty liver, yes/no**	35/28 (55.6/44.4)	34/36 (48.6/51.4)	0.421
**HCV RNA ^** a**^(log10), IU/mL **	6.05 (5.61-6.71)	5.69 (4.86-6.70)	0.288
**Peg-IFN α-2a/-α-2b ** ^**a**^	31/32 (49.2/50.8)	34/36 (48.6/51/4)	0.942
**HBV coinfection, yes/no** ****	5/58 (8.0/92.0)	6/64 (8.6/91.4)	0.894

^a^ Abbreviations: DM, diabetes mellitus; AST, aspartate aminotransferase; ALT, alanine aminotransferase; ALK-p, alkaline phosphatase; WBC, white blood cell count; INR, international normalized ratio; HCV, hepatitis C virus; peg-IFN, pegylated interferon

^b^ Heavy alcohol intake was defined as daily consumption > 50g before the antiviral therapy

^c^ Data of fibrosis stage was available only in the 30 patients undergoing liver biopsy. F1–F4 represent fibrosis scores, according to the METAVIR scoring system

**Table 2.** Treatment Profile and Virological Response of 133 Patients Stratified by Genotype. Data are Expressed as Either Median (25th–75th percentile values) or Number (Percentage, with 95% Confidence Interval for Response Rates)

	Genotype 1, (n = 63), No. (%)	Genotype 2, (n = 70), No. (%)	P value
**Average dose of peg-IFN α **			
peg-IFN α-2a, μg/week ^a^	180 (180-180), n = 31	180 (180-180), n = 34	0.340
peg-IFN α-2b, μg/kg/week	1.54 (1.37-1.69), n = 32	1.49 (1.39-1.65), n = 36	0.517
**Average dose of ribavirin, mg/Kg/day**	15.48 (14.11 - 17.04)	13.90 (12.24 - 15.13)	< 0.001
**80/80/80 adherence, No. (%)**	58 (92.1)	61 (87.1)	0.408
**Response to therapy, No. (%)**			
Early virological response	62 (98.4, 95.3-100)	70 (100, 100-100)	0.474
End-of-treatment virological response	61 (96.8, 92.5-100)	69 (98.6, 95.8-100)	0.603
Sustained virological response	53 (84.1, 75.1%-93.1)	63 (90, 83.0-97.0)	0.207
Relapse	8 (13.1, 4.6-21.6)	6 (8.7, 2.0-15.4)	0.417

^a^ Abbreviation: Peg-IFN, pegylated interferon

To identify factors related to relapse, baseline characteristics, treatment profiles, and adherence were compared between the patients who relapsed and those who achieved an SVR (Table 3). Univariate analysis revealed that lower AST (P = 0.010) and ALT levels (P = 0.003), use of Peg-IFN-α-2b (P = 0.048), HVL (P = 0.004), and unfavorable patterns of ALT change (P = 0.041) were significantly related to virologic relapse. Doses of RBV, including mean dose throughout the 24 weeks, before week 12, after week 12, the maximal reduction ratio, and 80% adherence were not significantly related to relapse. Multivariate logistic regression analysis documented HVL (P=0.015; odds ratio [OR] 14.754; 95% confidence interval (C.I.) 1.671–130.240), and unfavorable patterns of ALT change (P = 0.039; OR = 4.397; 95% C.I. 1.078-7.930) as independent factors related to virologic relapse.

**Table 3.** Factors Related to Relapse in Patients with Rapid Virologic Response by Univariate and Multivariate Analysis

Factors	Univariate Analysis			Multivariate Analysis		
	SVR (n = 116)	Relapse (n = 14)	P value	P value	OR ^a^	95% CI ^a^
**Age, y, mean ± SD**	53 ± 12	57 ± 11	0.312			
**Gender, male/female**	63/53	4/10	0.091	0.073		
**Heavy alcohol intake, yes/no**	12/104	1/13	1.000			
**BMI, mean ± SD, kg/m2**	24.78 ± 3.61	25.44 ± 2.77	0.344			
**Albumin, mean ± SD, g/dL**	4.3 ± 0.3	4.3 ± 0.3	0.806			
**Total bilirubin, mean ± SD, mg/dL**	0.8 ± 0.5	0.7 ± 0.2	0.595			
**AST **^ a^** , mean ± SD, U/L **	102 ± 70	58 ± 24	0.010	0.500		
**ALT ^** a**^ , mean ± SDU/L**	188 ± 132	96 ± 60	0.003	0.718		
**WBC ^** a**^ counts, mean ± SD, /mm**	5877 ± 1504	6196 ± 1788	0.612			
**Hemoglobin, mean ± SD, g/dL**	14.6 ± 1.7	14.0 ± 1.5	0.318			
**Platelet, mean ± SD, 10** ^** 3**^ **/μL**	188 ± 56	173 ± 61	0.255			
**APRI , mean ± SD** ^**a**^	1.55 ± 1.30	0.96 ± 0.55	0.149			
**Fibrosis stage, F1/2/3/4 ** ^** c**^	1/19/7/1	0/0/1/0	0.437			
**Cirrhosis, yes/no **	7/109	0/14	1.000			
**Fatty liver, yes/no**	61/55	7/7	0.855			
**VL , high/low** ^** a,b**^	62/54	13/1	0.004	0.015	14.754	1.671-130.240
**HCV ** ^**a**^ **genotype-1/2 **	53/63	8/6	0.572			
**Peg-IFN -α-2a/ -α-2b ** ^**a**^	59/57	3/11	0.048	0.121		
**Peg-IFN-α ≥/< 80% expected dose **	111/5	14/0	1.000			
**RBV ** ^**a**^ **dose ≥/< 80% expected dose **	105/11	11/3	0.176			
**Treatment ≥/< 80% expected duration **	116/0	14/0				
**80/80/80 adherence, yes/no**	105/11	11/3	0.176			
**Mean RBV dose, mean ± SD**	14.98 ± 4.32	14.80 ± 4.22	0.484			
**RBV dose in initial 12 weeks, mean ± SD **	16.10 ± 7.33	15.29 ± 3.70	0.856			
**RBV dose after week 12, mean ± SD **	13.89 ± 2.80	14.31 ± 4.82	0.226			
**Ratio of maximal reduction of RBV dose, mean ± SD **	14.01 ± 15.73	11.90 ± 21.11	0.226			
**Patterns of ALT change, unfavor/favor**	40/76	9/5	0.041	0.039	4.397	1.078-17.930

^a^ Abbreviations: AST, aspartate aminotransferase; ALT, alanine aminotransferase; APRI, AST-platelet ratio index; DM, diabetes mellitus; Peg-IFN, pegylated interferon; RBV, ribavirin; HCV, hepatitis C virus; WBC, white blood cell count; VL, baseline viral load; OR, odds ratio; CI, confidence interval

^b^ High viral load was defined as baseline HCV-RNA ≥600000 IU/mL, and low viral load was defined as HCV-RNA < 600000 IU/mL

^c^ Data of fibrosis stage was available only in the patients undergoing liver biopsy. F1–F4 represent fibrosis scores, according to the METAVIR scoring system

**Figure 1.**
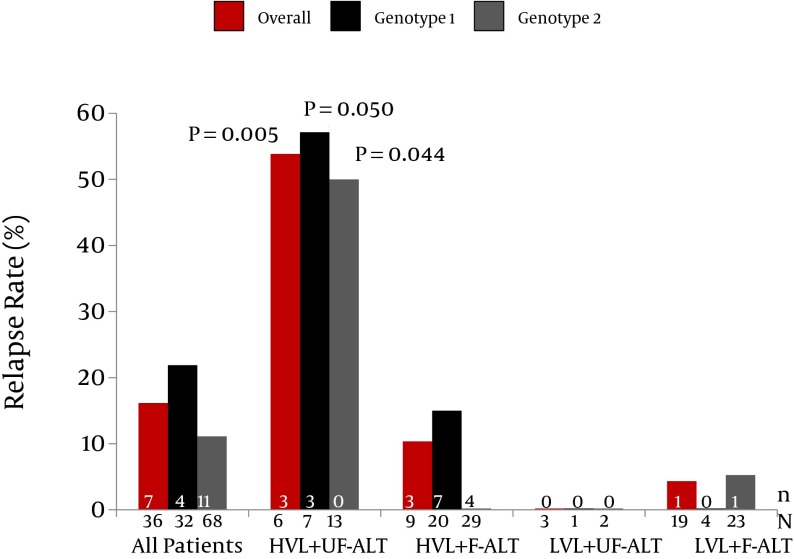
Relapse Rates of the 68 Patients Receiving Peg-IFN-α-2b Who Achieved an RVR and ETVR Stratified by Genotype, Viral Load, and the Pattern of ALT Change n, number of the relapsers; N, number of the patients receiving antiviral therapy; HVL, high viral load; F-ALT, favorable patterns of ALT change; UF-ALT, unfavorable patterns of ALT change

Baseline characteristics of the patients, including age (P = 0.483), gender (P = 0.679), BMI (P = 0.091), ALT (P = 0.879), genotypes (P = 0.955), presence of diabetes mellitus (P = 0.712), sonographic fatty liver (P = 0.870), and cirrhosis (P = 0.482) were not associated with the patterns of ALT change. Instead, use of Peg-IFN-α-2a was strongly associated with unfavorable patterns of ALT change (53.8%) compared to using Peg-IFN-α-2b (23.5%, P < 0.001). In patients receiving Peg-IFN-α-2b, unfavorable patterns of ALT change predicted relapse significantly in G-1 (50% vs. 12.5%, P = 0.047), G-2 (37.5% vs. 3.6%, P = 0.028), and all (43.8% vs. 7.7%, P = 0.002) patients. These patients were divided according to baseline viral loads and patterns of ALT change, then stratified according to genotypes, and their relapse rates were illustrated in Figure 1. In the patients with LVL, only one patient experienced relapse. In the patients with HVL, those with unfavorable patterns of ALT change had significantly higher relapse rates than those with favorable ones (53.8% vs. 10.3%, P = 0.005). The difference in relapse rates was attributable to both G-1 and G-2 infected subgroups (57.1% vs. 15%, P = 0.050 in the G-1, and 50% vs. 0%, P = 0.044 in the G-2 subgroup). In contrast, the subgroup receiving Peg-IFN-α-2a had an overall relapse rate of 4.8%, without significant differences between those with either favorable/unfavorable patterns of ALT changes (3.4% vs. 6.1%, P = 1.000) or G-1/-2 (3.4% vs. 6.1%, P = 1.000). Patients with HVL had higher VR rates (9.1%) compared to those with LVL (0%), but the difference was not significant P = 0.096).

## 5. Discussion

Achieving an RVR is highly predictive of SVR in patients with CHC receiving IFN-based antiviral therapy. Unfortunately, a substantial group of patients experience VR, especially if treatment is abbreviated. HVL is the most well-known factor to predict lower SVR rates (i.e. higher relapse rates). To date, other factors that impact relapse have rarely been evaluated. In addition to HVL, this study found that unfavorable patterns of ALT change (i.e., an increasing trend of ALT between weeks 4 and 12 and/or between weeks 12 and 24 [end-of-treatment]) after HCV-RNA clearance was also independently related to VR, especially in the subgroup with HVL, regardless of G-1 or G-2 status, receiving Peg-IFN-α-2b.

The strong prediction power of baseline VL, one of the most important factors affecting response of antiviral therapy, was shown in both genotypes in the present study. Nearly all patients who relapsed had a HVL. The only exception was a 70 year-old female patient with G-2 and baseline HCV-RNA level of approximately 535000 IU/mL. That patient’s treatment course was complicated by severe anemia (the lowest hemoglobin level was 6.9 g/dL), which led to use erythropoietin, and a reduction of RBV from 800 mg/day to 600 mg/day in the first half of treatment and a further decrease to 400 mg/day in the latter half of treatment.

Previously, VR in patients with CHC receiving combination therapy was attributable to the inability of commonly used assays to detect minimal residual viremia and reinvasion of virus from extrahepatic reservoirs (26). Basso et al. conducted a retrospective analysis on ALT elevations after serum HCV-RNA negativity achieved in patients with CHC undergoing combination therapy. That group concluded that elevations in ALT late in the course of treatment were related to VR. From that study, patterns of ALT change were derived (13). In the present study, elevation of ALT beyond the upper limit of normal after week 4 of treatment was shown to be an independent factor related to VR in patients achieved an RVR. The authors of the current study concur with Basso et al. who hypothesized that subclinical viral activity with minimal residual viremia, which was difficult to detect via commonly used HCV-RNA assays, might contribute to ALT elevations in the later phases of treatment. In addition, reinvading circulation of the hepatitis C virus from extrahepatic reservoirs may be inconstant; therefore, simply being unable to detect HCV-RNA at only a few time points (e.g. weeks 4, 12, and EoT) may not guarantee “persistent negativity” in the plasma. Alternatively, elevations of ALT may reflect hepatocyte damage caused by subclinical viral activity.

Undoubtedly abnormal on-treatment ALT levels may be attributed by factors other than subclinical viral activity. Background characteristics, including pretreatment ALT levels, BMI, body weight, and steatosis were associated with either ALT abnormalities or elevations during combination therapy (27, 28). In turn, hepatic steatosis and insulin resistance may affect response to combination therapy (29). Although higher levels of BMI were not significantly associated with either abnormal levels or increasing patterns of ALT in the study by Basso et al. and the presented study, further studies including measurements of insulin resistance or grading of steatosis may be helpful to more fully assess the impact of steatosis and/or subclinical viral activity on VR. Another factor contributing to on-treatment abnormal ALT levels described by Aoki et al. was using Peg-IFN-α-2a. That factor was strongly associated with unfavorable patterns of ALT in the present study, even though the inclusion criteria and definitions of on-treatment abnormal ALT levels were somewhat different between the current study and that of Aoki et al. (27). Higher molecular weight of Peg-IFN-α-2a compared to Peg-IFN-α-2b, leading to longer half-life, was supposed to account for higher on-treatment ALT levels (27).

**Figure 2.**
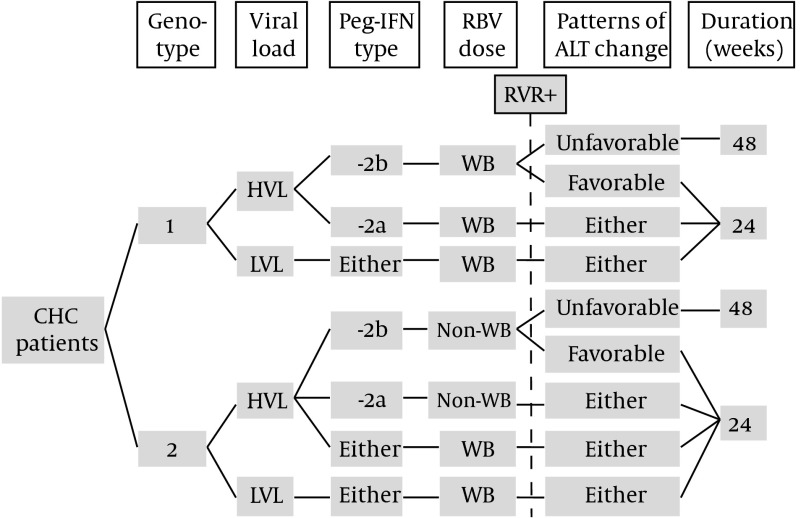
Proposed Algorithm With Modified Durations for Peg-IFN and RBV Combination Therapy in Patients With Chronic Hepatitis C Attaining RVR HVL, baseline high viral load; LVL, baseline low viral load; WB, weight-based (dose of RBV)

Further subgroup analysis disclosed that the VR rate in patients receiving Peg-IFN-α-2a was considerably low, even in the G-1 infected patients with HVL (9.1%), which concurred with the data from Yu et al. (11.1%) (4). Among the patients treated with Peg-IFN-α-2a, the role of genotype, viral load, and patterns of ALT change in predicting VR were limited; however, combining HVL and unfavorable patterns of ALT change could identify a high-risk subgroup for VR (~50%) in patients receiving Peg-IFN-α-2b, regardless of genotype status. Those results suggest that 48 weeks of therapy should be offered to G-1, HVL patients with unfavorable patterns of ALT receiving combination therapy with Peg-IFN-α-2b, even if they achieve an RVR. Forty-eight weeks of therapy should also be considered for G-2, HVL patients who achieve an RVR, but have unfavorable patterns of ALT, even though current treatment guidelines still recommend a 24 week treatment course. Despite extended therapy, prescription with weight-based RBV since the start may overcome the “low responsiveness” resulting from factors such as HVL, advanced fibrosis, or insulin resistance (1). In a study conducted by Yu et al. patients with G-2 HCV who received weight-based RBV and attained an RVR, could achieve extremely high SVR rates (97.7–100%) (12). That superior response rate was considered to be contributed by higher RBV doses (mean dose was 15.3 mg/kg/day) in contrast to the 9.52 mg/kg/day in the ACCELERATE trial, which reported an SVR rate of 85% in G-2 patients with RVR (12, 30 ). In contrast, an abbreviated 24 week course prescribed to patients with G-1, HVL, attaining RVR, and with favorable patterns of ALT during combination therapy with Peg-IFN-α-2b may be sufficient. An explosive increase in the expenditure of antiviral therapy for CHC is expected in the near future, because current practical guideline has included direct antiviral agents in the first-line regimen (31). Therefore, it is important to identify some “super-responders” whose antiviral therapies could be simplified and/or abbreviated, if possible, without compromise to efficacy, to reduce the growth of expenditure, and to avoid unnecessary adverse effects. Based on the aforementioned subgroup analyses, an algorithm, including genotype, baseline VL, treatment drugs, and patterns of ALT change was proposed by the authors of this study for patients with CHC attaining RVR (Figure 2).

RBV doses, including lower total doses, mean doses, and greater reductions in dose, were reported to predict VR in some studies (16, 17, 19). In the current study, mean dose throughout the 24 weeks, in the initial and last 12 weeks, maximal reduction ratios, and 80% adherence were all examined; however, no significant association was identified to relapse. This study could not negate the influence of RBV dose on relapse because of the relatively small sample size. The supposition that reductions in RBV dose contribute less in “easy-to-treat” patients (such as those included in the presented study) than that in the general population of CHC patients’ needs to be more fully evaluated. Further, determination of IL-28B gene polymorphism, another factor strongly related to SVR, was not available in the present study due to the retrospective nature of the study. Either favorable or unfavorable alleles of both the rs12979860 and rs8099917 polymorphism reportedly have comparable SVRs or relapse rates in patients with CHC attaining an RVR during the combination therapy; therefore, the role of IL-28B gene polymorphism on VR in patients attaining RVR may be limited (32-35).

There are several study limitations as follow. First, this was a retrospective study, and further prospective studies are warranted to verify the determinant power of patterns of ALT change. Second, a relatively small sample size may have influenced the statistical power for comparison. Third, hepatic fibrosis, insulin resistance, and steatosis, which were previously shown to be related to SVR, were not exactly assessed in the present study. Further studies including these factors are certainly warranted. Fourth, the patterns of ALT change were determined by ALT levels only at weeks 4, 12, and 24, which clearly did not reflect transient elevations between those selected time points. Modifications to the definitions of ALT change patterns may be warranted to better reflect subclinical viral activity in the liver.

In conclusion, VR occurred infrequently in the included group of patients with CHC attaining RVR during the combination therapy, regardless of genotype. HVL and unfavorable patterns of ALT change were independent factors predicting VR. Based on stratified subgroup analysis, patients with LVLs can likely be treated with only 24 weeks of therapy, with only a minimal chance of relapse. In patients with HVLs, 24 weeks of therapy can be used reliably in patients receiving Peg-IFN-α-2a and in patients receiving Peg-IFN-α-2b with favorable patterns of ALT change, once attaining a RVR. Further prospective studies are warranted to validate recommendations presented herein.
